# Homonymous visual field defect and retinal thinning after occipital stroke

**DOI:** 10.1002/brb3.2345

**Published:** 2021-09-06

**Authors:** Avan Sabir Rashid, Darian Rashid, Ge Yang, Hans Link, Helena Gauffin, Yumin Huang‐Link

**Affiliations:** ^1^ Division of Neurology, Department of Biomedical and Clinical Sciences, Faculty of Medicine and Health Sciences Linköping University Linköping City Sweden; ^2^ Institute of Ophthalmology University College London London UK; ^3^ Department of Clinical Neuroscience Karolinska Institute Stockholm Sweden

**Keywords:** ganglion cell and inner plexiform layer, homonymous visual field defect, occipital stroke, optical coherence tomography, retinal nerve fiber layer

## Abstract

**Introduction:**

Stroke is the most common cause of homonymous visual field defects (VFD). About half of the stroke patients recover from VFD. However, relationship between VFD and retinal changes remains elusive.

**Purpose:**

To investigate the association between occurrence of VFD, changes of macular ganglion cell and inner plexiform layer (GCIPL) and its axon retinal nerve fiber layer (RNFL) detected with optical coherence tomography (OCT).

**Patients and methods:**

The study consists of retrospective review of medical records and follow‐up examinations. Patients with acute occipital stroke were registered. VFD was identified with confrontation and/or perimetry tests at the onset. At follow‐up, the patients were examined with visual field tests and OCT measurements.

**Results:**

Thirty‐six patients met the inclusion criteria. At onset, 26 patients (72%) had VFD. At follow‐up >1 year after stroke, 13 patients (36%) had remaining VFD: 5 had homonymous hemianopia, 5 had homonymous quadrantanopia, and 3 had homonymous scotomas. Average thickness of GCIPL and RNFL were significantly reduced in each eye in patients with VFD compared to non‐VFD (NVFD) (*p* < .01 for all comparisons). Thickness of superior and inferior RNFL quadrants was significantly reduced in VFD compared to NVFD (*p* < .01 for both). Among these 13 patients, 4 had characteristic homonymous quadrant‐GCIPL thinning, 2 had characteristic homonymous hemi‐GCIPL thinning, and 7 had diffuse GCIPL thinning.

**Conclusion:**

GCIPL and RNFL thinning were observed in the patients with VFD. GCIPL thinning appears in two forms: atypical diffuse thinning, or homonymous hemi‐GCIPL thinning. Examining GCIPL and RNFL provides easy and reliable objective measures and is therefore proposed to be of predictive value on visual function.

## Graphical abstract

GCIPL and RNFL thinning was observed in the patients with VFD. GCIPL thinning appears in two forms: atypical diffuse thinning, or homonymous hemi‐GCIPL thinning. Examining GCIPL and RNFL provides easy and reliable objective measures to predict visual function.

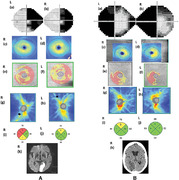

## 1 INTRODUCTION

Homonymous visual field defect (VFD) involves visual fields on the same side of both eyes resulting from damages of retrochiasmal visual pathways. Lesions are commonly localized in cerebral visual (occipital) cortex and optic radiation (Kedar et al., 2007; Zhang et al., 2006b). Homonymous VFD is often disabling, causing difficulties with reading and visual scanning (Goodwin, 2014; Tharaldsen et al., 2020). This condition can cause collisions with approaching objects, obstacles or cars (Goodwin, 2014; Tharaldsen et al., 2020). In clinical scenarios, characteristics of visual field abnormalities (pattern, form, size, congruity) corresponding to anatomic structure and physiological functions are traditionally used for localizing pathologic brain lesions (Prasad & Galetta, 2012). Retrochiasmal VFD is most commonly caused by stroke (Dinkin, 2017; Räty et al., 2018; Zhang et al., 2006a). Spontaneous recovery mostly occurs within 3 months after stroke (Cassidy et al., 2001; Gray et al., 1989). Objective measures to predict permanent VFD and rehabilitation options are limited (Goodwin, 2014; Svaerke et al., 2019; Tharaldsen et al., 2020; Zhang et al., 2006c).

Transneuronal antegrade degeneration from the retina to the cerebral visual cortex has been demonstrated in glaucoma patients (Gupta et al., 2009) and patients with optic neuritis (ON) (Tur et al., 2016). Atrophy of the lateral geniculate nucleus and the optic radiation was detected with magnetic resonance imaging (MRI) in such patients. Transneuronal retrograde degeneration from the primary visual cortex to the optic tract has also been demonstrated with MRI in patients with occipital lobe lesions (Bridge et al., 2011; Millington et al., 2014). In addition, optical coherence tomography (OCT) has been recently used to quantitively investigate retrograde degeneration in the retina (Dinkin, 2017; Foster et al., 2019; Yamashita et al., 2012). Antegrade degeneration occurs due to the presynaptic input loss after death of presynaptic neurons in the retina. Retrograde degeneration is supposed to take place after loss of postsynaptic axonal transportation (Jindahra et al., 2009), but comparatively it is not as common as antegrade degeneration. Acquired transneuronal retrograde degeneration has not been well recognized in clinical practice. The diagnostic criteria of retrograde degeneration are not yet established. The pattern and form of retrograde degeneration in the retina as measured with OCT has been mainly based on case reports (Foster et al., 2019; Hokazono et al., 2019; Newman‐Wasser et al., 2019; Tatsumi et al., 2005). A few studies have shown that the retinal ganglion cell and inner plexiform layer (GCIPL) in the macula and their axons of retinal nerve fiber layer (RNFL) in the optic disc are reduced after retrochiasmal lesions (Jindahra et al., 2012; Keller et al., 2014; Park et al., 2013; Yamashita et al., 2016). However, unanimous pattern and form of GCIPL and RNFL changes during retrograde degeneration are still unclear. Whether these changes are associated with permanent VFD need to be addressed.

In this study, we reviewed medical records of patients with occipital stroke and identified patients with or without VFD during the acute phase. At follow‐up, OCT was used to examine changes of GCIPL and RNFL, and to quantify their thickness. The relationship between OCT measures and VFD were analyzed. The predictive value of OCT measures for VFD was evaluated.

## 2 METHODS

### 2.1 Patient selection

Two investigators reviewed the medical records of all patients with acute occipital stroke who were admitted within 1 week after onset to the Stroke Unit, Division of Neurology at Linköping University Hospital, Sweden. The inclusion period was between January 2017 and December 2018 and the timing for follow‐up was at least 1 year after onset. This was based on the fact that occurrence of transneuronal retrograde degeneration takes at least 1 year (Dinkin, 2017) and spontaneous improvement VFD occurs after 6 months (Zhang et al., 2006c). Patients with severe cognitive or physical impairment, glaucoma, macular degeneration, diabetic retinopathy, or other eye diseases were excluded from the study.

Data from onset to follow‐up of the 36 patients who met the inclusion criteria were available: 2 patients went through brain MRI, 3 had computed tomography (CT), and 31 went through both MRI and CT at admission.

Patients’ medical records related to stroke are electronic. The Regional Ethics Committee of Linköping University for clinical research approved the study (2020‐00046).

### 2.2 Ophthalmological and visual field examination

Visual field test was performed in all patients at admission by at least two neurologists using confrontation visual field examination. In case of discrepancy or uncertainty, Humphrey visual field test was applied at admission and performed by an ophthalmologist.

At follow‐up, pupil size with light reflexes, eye movements, fundus of the eyes, and visual field by confrontation test were performed on all patients. Digital funduscopy (Visuscout 100, Carl Zeiss Meditec AG, Jena, Germany) was used in all patients. Visual field by Humphrey perimetry was applied according to the clinical planning rather than research timing. Those patients who had one of the following findings were examined by perimetry: (1) abnormal or uncertain confrontation test, or (2) subjective visual disturbances, or (3) abnormal OCT measures. The program used in Humphrey automated perimetry was Faster Swedish Interactive Thresholding Algorithm (faster SITA).

The patients were divided into two groups according to results from visual field testing: (1) homonymous VFD and (2) non‐VFD (NVFD).

### 2.3 Optical coherence tomography

At follow‐up, all patients underwent examination with spectral‐domain OCT (SD‐OCT, Cirrus 4000, Carl Zeiss Meditec, software version 6.5, CA, USA). Retinal imaging was obtained according to the standard protocol (Schippling et al., 2015). Macular GCIPL thickness was measured using macular cube 512 × 128 protocol with a 6 mm rim centered at the fovea. Peripapillary RNFL thickness was measured using optic disk 200 × 200 protocol with a custom 3.4 mm ring centered at the papilla. The retinal thickness deviation map was generated automatically using the built‐in software. The yellow color indicates retinal thickness <5% of the normative level and the red indicates <1% of the normative level. The average thickness of RNFL and of four RNFL quadrants: superior (S), inferior (I), temporal (T), and nasal (N), were registered from each eye. Scans with signal strength of 7/10 or above were included.

### 2.4 Statistical analyses

Statistical analyses were completed on statistical package for social science (SPSS) version 26 (SPSS Inc.). Data from each eye were analyzed separately to avoid bias of inter‐eye correlation. Normality was analyzed using Shapiro‐Wilk test. The mean and the standard deviation or the median, 25th and 75th percentiles (Q1 and Q3: interquartile range, IQR) were used for continuous variables, and the percentages for categorical variables. Depending on data normality distribution, Student's *t*‐test or Mann–Whitney *U*‐test were used to compare continuous data between the study groups, and chi‐squared test or Fisher's exact test for categorical data between two groups was considered statistically significant (*p* ≤ .05).

## 3 RESULTS

### 3.1 Clinical data

Thirty‐six patients with occipital stroke met the study criteria and were enrolled in the study. Thirty‐three of them had cerebral infarction and three had cerebral hemorrhage. Data from the extraction of medical records revealed that 26 (72.2%) of 36 patients had VFD at the onset tested by confrontation visual field examination. In case of discrepancy or uncertainty of OCT or confrontation test, perimetry Humphrey was applied. At the follow‐up, 13 (36%) of 36 patients had remaining VFD, 23 (64%) showed normal VF.

The demographic data from these two groups of 23 patients with NVFD and 13 with VFD are shown in Table 1. There was no significant difference between the two groups regarding age, sex, stroke risk factors, NIH stroke scale (NIHSS) at the onset, acute therapy with intravenous thrombolysis, or previous stroke prevention therapies. The majority of patients were men in both groups. Pre‐existing hypertension was the most common risk factor, followed by atrial fibrillations in both groups. NIHSS at 3 month follow‐up was higher in VFD than in NVFD group (*p* = .02).

**TABLE 1:** Clinical data of the 36 patients in two groups based on visual field defect

	NVFD *n* = 23	VFD *n* = 13	*p‐*Value
Age, mean ± SD	67.3 ± 9.0	70.4 ± 15.3	.51
Sex (%)			.44
Female	5 (21.7)	5 (38.5)	
Male	18 (78.3)	8 (61.5)	
Duration (months), mean ± SD	24.3 ± 5.6	24.2 ± 5.8	.96
Risk factors (%)			
Hypertension	14 (60.9)	7 (53.8)	.68
Atrial fibrillation	11 (47.8)	3 (23.1)	.14
Diabetes	5 (21.7)	2 (15.4)	1.0
Previous stroke	5 (21.7)	1 (7.7)	.39
Previous TIA	3 (13)	0 (0)	.29
NIHSS, median (IQR)			
At onset	2 (0‐4.3)	2 (1‐4)	.58
At follow‐up, 3 m	0	1 (0‐1.5)	.02*
IV thrombolysis (%)	3 (13)	1 /7.7)	1.0
Lesion location (%)			.97
Occipital	9 (39.1)	5 (38.5)	
Occipital+	14 (60.9)	8 (61.5)	
Therapy at onset (%)			
Anti‐platlet	9 (39.1)	4 (30.8)	.73
Anti‐coagulantia	1 (4.3)	1 (7.7)	1.0
Anti‐hypertensive	13 (56.5)	7 (53.8)	.88
Anti‐lipids	11 (47.8)	6 (46.1)	.92
Anti‐diabetes	3 (13.0)	1 (7.7)	1.0

Abbreviations: IQR, interquartile range; IV, intravenous; NIHSS, National Institutes of Health Stroke Scale; NVFD, non‐visual field defect; occipital+, occipital lobe plus other lobes; SD, standard deviation; TIA, transient ischemic attack; VFD, visual field defect.

*p ≤ .05 using Mann–Whitney *U*‐test.

### 3.2 Visual field defects

At follow‐up, 13 of 36 patients (36%) were identified to have VFD: 5 had homonymous hemianopia, 5 had homonymous quadrantanopia, and 3 had homonymous scotomas. Eleven of 13 patients had VFD without improvement since stroke onset, 2 of these 13 patients had remaining VFD but with improvement: 1 with homonymous hemianopia and 1 with homonymous quadrantanopia at the onset, both had homonymous scotomas at follow‐up. Ten of 36 patients showed normal visual fields at both onset and follow‐up. Thirteen of 26 patients with VFD at the onset had completely recovered at 1 year follow‐up. All 13 patients with remaining VFD and 9 of the 23 with NVFD patients were examined by Humphrey perimetry at follow‐up. Remaining 14 of those 23 NVFD patients did not go through Humphrey examination according to the clinical planning at follow‐up, while 7 of them were examined by Humphrey perimetry at onset with normal findings.

Out of the 13 patients with VFD, 6 were was identified with confrontation test and Humphrey perimetry, 7 was identified only by Humphrey perimetry, but not by the confrontation test. Nine patients with NVFD showed consistent normal results of confrontation test, perimetry, and OCT.

### 3.3 Association of GCIPL and RNFL thickness with VFD

The average thickness of the GCIPL of the right and left eyes was significantly thinner in the VDF group compared with the NVDF group (Table 2) (*p* < .01 for each eye). The average thickness of the RNFL was also significantly thinner on the right and left eyes in the VDF group compared with the NVDF group (Table 2) (*p* < .01 for each eye).

**TABLE 2:** The thickness of RNFL and GCIPL (μm) in two groups, based on visual field defect

	VFD		NVFD	
Thickness (μm)	Right eye	Left eye	Right eye	Left eye
RNFL (*n*)	13	12	23	21
Average	78.9 ± 8.1	77.3 ± 7	89 ± 6.7**	88 ± 7.4**
Quadrants				
Superior	86 (77–97.5)	89 (85–103.5)	101 (95–114)**	103 (99–122)**
Inferior	99.3 ± 14.6	96.8 ± 13.1	115.9 ± 11.9**	114.1 ± 13.4**
Temporal	57.2 ± 16.5	57.2 ± 17.8	62.4 ± 13.4	60.7 ± 10.1
Nasal	71 (66–77.5)	64 (62.5–72.5)	70 (65–76)	67 (62.5–75.5)
GCIPL (eyes)	12	12	22	20
Average	61.5 (50–67.8)	64.5 (62–71.3)	81 (74.8–84)**	78 (74.5–83.8)**

Abbreviations: GCIPL, ganglion cell and inner plexiform layer; IQR, interquartile range; NVFD, non‐present visual field defect at examination; RNFL, retinal nerve fiber layer; SD, standard deviation; VFD, visual field defect.

**p ≤ .01 using Student's *t*‐test for normally distributed data or Mann–Whitney *U*‐test for non‐normally distributed data.

**FIGURE 1:**
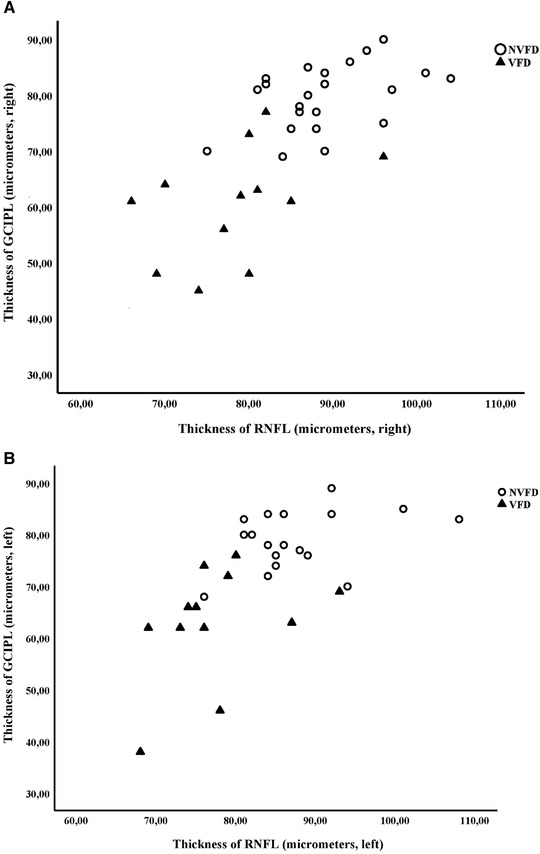
Mean thickness of GCIPL and RNFL at follow‐up after stroke in patients with or without visual field defect (VFD or NVFD). In the VFD group, the average thickness of GCIPL and RNFL is significantly reduced in the right (A) as well as in the left eye (B) compared to the NVFD group (*p* < .01 for each eye). Mann–Whitney *U‐*test was applied for GCIPL analysis, and Student's *t*‐test was applied for RNFL analysis, based on normality analysis Abbreviations: GCIPL, ganglion cell and inner plexiform layer; NVFD, non‐visual field defect; RNFL, retinal nerve fiber layer; VFD, visual field defect. *p ≤ .05; **p ≤ .01.

The thickness of GCIPL was correlated with the thickness of RNFL in each eye (right 0.67, left 0.62, Pearson correlation, *p* < .01 for each eye) (Figure 1A,B).

**FIGURE 2:**
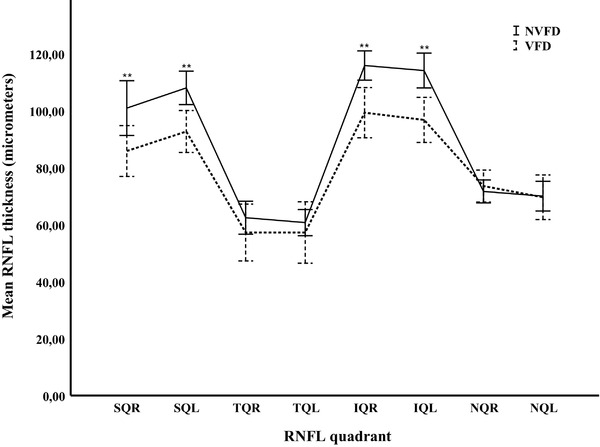
Mean thickness of RNFL quadrants at follow‐up after stroke in patients with (VFD) or without visual field defect (NVFD). The thickness of RNFL quadrants was analyzed in each eye. Significant reduction of RNFL thickness was observed in the superior (*p* < .01 for each eye) and inferior (*p* < .01 for each eye) quadrants in the VFD group (dotted line) compared to the NVFD group (solid line). The thickness of RNFL temporal and nasal quadrants showed no significant difference between the two groups. Vertical bars represent 95% confidence intervals Abbreviations: IQL, inferior quadrant left; IQR, inferior quadrant right; NQL, nasal quadrant left; NQR, nasal quadrant right; NVFD, non‐visual field defect; RNFL, retinal nerve fiber layer; SQL, superior quadrant left; SQR, superior quadrant right; TQL, temporal quadrant left; TQR, temporal quadrant right; VFD, visual field defect. *p ≤ .05 **p ≤ .01.

The significant reduction of RNFL thickness in the VFD group when compared with the NVFD group was confined to the superior and inferior quadrants of the optic disc (Figure 2, Table 2) (*p* < .01 both quadrants of each eye), but not to the temporal and nasal quadrants.

### 3.4 Two forms of GCIPL thinning in the patients with VFD

**FIGURE 3:**
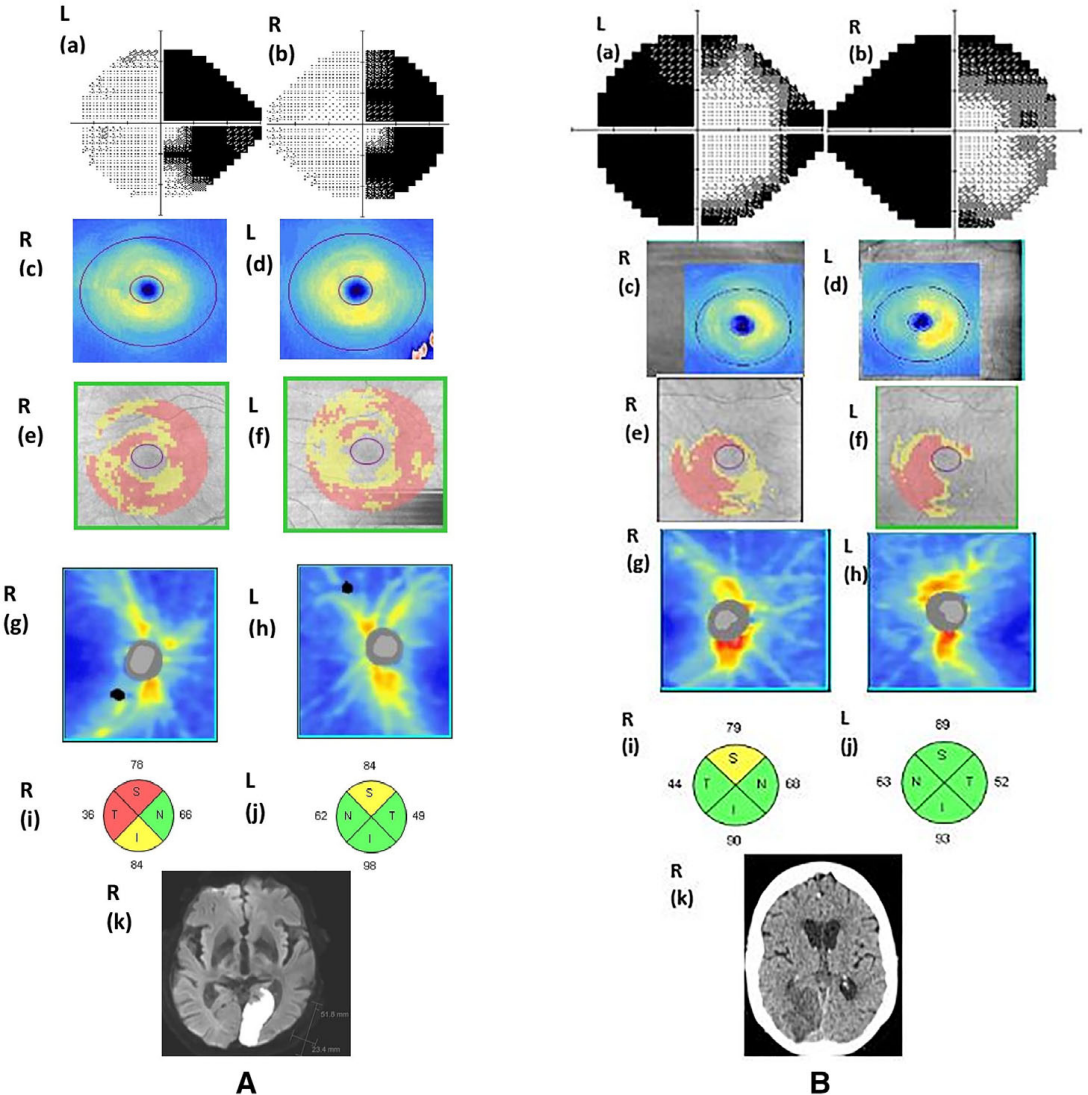
(A) An 80‐year‐old male with diffuse GCIPL thinning and homonymous hemianopia. (a,b) Right side homonymous hemianopia with macular sparing detected by Humphrey visual field test; (c,d) diffuse GCIPL thinning on the OCT thickness map; (e,f) diffuse GCIPL thinning with tendency of left homonymous thinning on the OCT deviation map; (g,h) RNFL thinning on the thickness map; (i) reduced right RNFL superior, temporal and inferior quadrants (yellow coded: outside of the 95% normal limit; red coded: outside of the 99% normal limit); (j) reduced left RNFL superior quadrant (yellow coded); (k) an acute cerebral infarction with hyperintensity in the left occipital lobe as shown by diffusion MRI. (B) An 88‐year‐old female with homonymous GCIPL thinning and contralateral homonymous hemianopia. (a,b) Left side homonymous hemianopia detected by Humphrey visual field test; (c,d) right side homonymous hemi‐GCIPL thinning on the OCT thickness map; (e,f) right side homonymous hemi‐GCIPL thinning on the OCT deviation map; (g,h) relatively preserved RNFL thickness except (i) reduced thickness in the right superior RNFL quadrant (yellow coded: outside of the 95% normal limit); (j) normal left RNFL thickness in all quadrants (green coded); (k) a subacute cerebral infarction in the right occipital lobe shown by CT

Seven patients with VFD (53.8%) had diffuse GCIPL‐thinning (Figure 3A): 2 of them showed homonymous scotoma, 2 had homonymous quadrantanopia, and 3 showed homonymous hemianopia. Four patients with VFD (30.8%) had characteristic homonymous quadrant‐GCIPL thinning: 3 of them showed homonymous quadrantanopia and 1 showed homonymous scotoma. Two patients with VFD (15.4%) had characteristic homonymous hemi‐GCIPL thinning: both of them showed homonymous hemianopia. Homonymous GCIPL thinning is corresponding to ipsilateral occipital lesions and contralateral homonymous quadrantanopia or hemianopia (Figure 3B). There was no patient in the NVFD group who showed homonymous hemi‐ or quadrant‐GCIPL thinning.

## 4 DISCUSSION

This study demonstrates that in patients with VFD after occipital stroke, the macular ganglion cells in the GCIPL and their axons in the RNFL are significantly affected compared to those with NVFD as measured by OCT. In patients without VFD after occipital stroke, the GCIPL and RNFL are relatively preserved compared to those with VFD. Tansneuronal retrograde degeneration of the visual pathway after stroke has been mostly observed in small case series or case reports in patients with homonymous VFD (Keller et al., 2014; Millington et al., 2014; Park et al., 2013; Yamashita et al., 2016). However, the extent and pattern of the retrograde degeneration of the visual pathway after occipital stroke is unclear, and the association between VFD and retrograde degeneration remains elusive (Dinkin, 2017).

We present a series of patients with acute occipital stroke with or without VFD. The lesion location was verified by neuroimaging brain CT or MRI, and most patients underwent both CT and MRI during the acute phase. The majority of the patients showed VFD at the onset, which is in agreement with observations from other studies (Räty et al., 2018; Tharaldsen et al., 2020; Zhang et al., 2006a). In our study, all the VFD were homonymous and the most common VFD are quadrant‐ or hemianopia at the onset. After more than 1 year of follow‐up, about 1/3 of the patients with occipital stroke had remaining homonymous VFD. This means that about half of the patients with initial VFD recovered spontaneously. A previous large case series report has observed spontaneous recovery in about 38% of patients (Zhang et al., 2006c). VFD caused by brain lesions often recovers spontaneously after 3 months or later, and rehabilitation options are limited (Goodwin, 2014; Räty et al., 2018; Reitsma et al., 2013; Schneider et al., 2019; Tharaldsen et al., 2020). In our study as improvement of VF, homonymous quadrant‐ or hemianopia transformed into homonymous scotomas. Spontaneous recovery from VFD can result from the plasticity of the visual cortex (Gilbert & Wu, 2012; Reitsma et al., 2013), though it is unclear to what extent and how large‐scale plasticity following damage to the human visual cortex occur. Recovery of VFD may also depend on reperfusion of penumbra, vanished edema over time, stimulations from the surroundings, and so on (Millington et al., 2014; Schneider et al., 2019).

At the follow‐up more than 1 year after stroke, there was a significant difference in OCT measures in patients with permanent VFD compared to those with NVFD. Average GCIPL thickness in each eye was reduced in the patients with VFD compared to those with NVFD. Reduced GCIPL presents in the form of atypical diffuse or focal incongruent thinning, and/or characteristic homonymous quadrant‐ or hemi‐GCIPL thinning. Atypical GCIPL thinning has also been observed in different disease groups including congenital damages, multiple sclerosis (MS) plaques, surgical resection of Meyer's loop/temporal lobe, and tumors in the retrochiasmal pathways (de Vries‐knoppert et al., 2019; Gabilondo et al., 2014). In addition, atypical GCIPL thinning can occur in eye disorders such as glaucoma and retinopathy of different pathologies. It is difficult to define such GCIPL thinning as a result of retrograde degeneration or lesions in the brain. The characteristic homonymous GCIPL thinning detected by OCT corresponds to ipsilateral occipital lesions and to contralateral homonymous quadrant‐ or hemianopia. This pattern of GCIPL thinning could be considered as direct evidence of retrograde degeneration and has been found in both congenital and acquired damages of the retrochiasmal pathway (Foster et al., 2019; Huang‐Link et al., 2014; Keller et al., 2014; Mühlemann et al., 2020; Yamashita et al., 2016). In our study, those with NVFD showed no homonymous GCIPL thinning and the average GCIPL thickness was relatively intact. These findings suggest that GCIPL is associated with visual function.

Looking further into the RNFL thickness reduction, reduced peripapillary RNFL as measured with OCT are common in patients with or without ON (Huang‐Link et al., 2015). Specifically, the largest reduction of RNFL thickness was observed in the temporal RNFL quadrant (Birkeldh et al., 2017). Longitudinal follow‐up suggested that temporal RNFL thinning is highly sensitive and associated with both cognitive and physical disability in MS. MS patients had significantly reduced temporal RNFL thickness. In our study, the average RNFL thickness is also reduced in patients with VFD compared to patients with NVFD. But the reduced RNFL was only confined to the superior and inferior quadrants, which agrees with the study by Park et al (Park et al., 2013). However, we did not find a difference in the thickness of the temporal and nasal RNFL quadrants between patients with VFD and patients with NVFD. In each eye, the thickness of temporal and nasal RNFL quadrants is similar and comparable in the two groups. The thinning of the superior and inferior quadrants may indicate future permanent VFD after stroke.

This study has some limitations. The sample size is small, especially with fewer patients with VFD. Patients with diabetes mellitus and hypertension without retinopathy were included in the study. This was based on the previous observations that RNFL thickness in patients with diabetes or hypertension but without retinopathy was not different from normal controls (Bhargava et al., 2012; Park et al., 2011). Moreover, the neuroradiological assessment was according to the clinical planning rather than research timing, and lesion localization and size in the occipital visual cortex are not detailed. However, information about GCIPL changes in these conditions is lacking (Gabriela et al., 2017; Lee et al., 2018). Finally, not all 23 patients with NVFD were examined with perimetry at follow‐up which may be a risk of circular reasoning for VFD and OCT findings. However, 9 of them have gone through perimetry with normal VF. Confrontation test is a reliable, convenient, and valuable tool in clinical practice and is recommended for screening of VFD, especially at stroke onset (Cassidy et al., 2001). But we might risk to miss those patients with central scotoma by using only confrontation test at the follow‐up (Townend et al., 2007).

Taken together, the majority of patients with occipital stroke have homonymous VFD at the onset. About 1/3 of these patients have permanent homonymous VFD 1 year after stroke. The patients with VFD show retinal atrophy secondary to GCIPL, and RNFL thinning. GCIPL thinning occurs in two forms: atypical diffuse thinning, or/and characteristic homonymous hemi‐GCIPL thinning ipsilateral to the occipital lesions and contralateral to VFD. RNFL thinning confined to superior and inferior quadrants of the optic disc is also observed in the patients with VFD compared to the patients without VFD or with temporary VFD. GCIPL and RNFL measures by OCT are highly sensitive and quantitative at a few micrometers level. Examining GCL and RNFL provides easy and reliable objective measures to predict and monitor visual function after occipital stroke.

## FUNDING INFORMATION

Faculty of Medicine and Health Sciences of Linköping University and County Council of Östergötland (project no. LIO 799111 and LIO 858051)

## AUTHOR CONTRIBUTION

Yumin Huang‐Link, Hans Link, and Ge Yang developed the theory and performed pilot study. Avan Sabir Rashid, Darian Rashid, and Yumin Huang‐Link designed the database, model and framework, and analyzed the data. They participated in both retrospective and follow‐up clinical work and data analyses. Helena Gauffin and Yumin Huang‐Link developed, financed, and supervised this work. Darian Rashid and Yumin Huang‐Link performed statistical analyses. Yumin Huang‐Link, Ge Yang, Darian Rashid, Avan Sabir Rashid, Hans Link, and Helena Gauffin contributed to the manuscript writing.

### PEER REVIEW

The peer review history for this article is available at https://publons.com/publon/10.1002/brb3.2345

## CONFLICT OF INTEREST

The authors declare no potential conflicts of interest with respect to the research, authorship, and/or publication of this article.

## Data Availability

Anonymized summary data on the patient cohort that support the findings of this study are available from the corresponding author upon reasonable request.
